# Testosterone improves the differentiation efficiency of insulin-producing cells from human induced pluripotent stem cells

**DOI:** 10.1371/journal.pone.0179353

**Published:** 2017-06-08

**Authors:** Haikun Liu, Dongsheng Guo, Aynisahan Ruzi, Yan Chen, Tingcai Pan, Fan Yang, Jialiang Li, Kecheng Xu, Tiancheng Zhou, Dajiang Qin, Yin-xiong Li

**Affiliations:** 1 Institute of Public Health, Guangzhou Institutes of Biomedicine and Health, Chinese Academy of Sciences, Guangzhou, China; 2 School of Life Sciences, Anhui University, Hefei, China; 3 Key Laboratory of Regenerative Biology, South China Institute for Stem Cell Biology and Regenerative Medicine, Guangzhou Institutes of Biomedicine and Health, Chinese Academy of Sciences, Guangzhou, China; 4 Guangdong Provincial Key Laboratory of Biocomputing, Guangzhou Institutes of Biomedicine and Health, Chinese Academy of Sciences, Guangzhou, China; 5 University of Chinese Academy of Sciences, Beijing, China; 6 Guangzhou Fuda Cancer Hospital, Guangzhou, China; NIDCR/NIH, UNITED STATES

## Abstract

Human induced pluripotent stem cells (hiPSCs) may provide potential resource for regenerative medicine research, including generation of insulin-producing cells for diabetes research and insulin production. Testosterone (T) is an androgen hormone which promotes protein synthesis and improves the management of type 2 diabetes in clinical studies. Concurrently, co-existed hyperandrogenism and hyperinsulinism is frequently observed in polycystic ovary syndrome, congenital adrenal hyperplasia and some of Wermer's syndrome. However, the relationship among androgens, insulin and the differentiation of pancreatic β cells is still not fully clear. Here we find that T improves the differentiation efficiency of insulin-producing cells from hiPSCs. The addition of T into routine differentiation formula for pancreatic β cells increases the differentiation efficiency from 12% to 35%. The administration of T promotes the expression of key genes associated with β cells differentiation including *NGN3*, *NEUROD1* and *INS*. This finding benefits the ongoing process to optimize the differentiation protocol of pancreatic β cells from hiPSCs, and provides some degree of understanding the clinical management of T for type 2 diabetes.

## Introduction

There were 382 million people living with diabetes by the end of 2013, and this number is expected to rise to 592 million by 2035 [1]. The majority of diabetes includes type 1 and type 2. Type 1 diabetes are insulin-dependent and considered mainly related with chronic autoimmune disorder resulting in pancreatic β cells are gradually destroyed [2]. Although the occurrence of hyperglycemia is inevitable in Type 1 diabetes as well as late stage of Type 2 diabetes, exogenous insulin is still the gold standard for these diabetic patients. Cells-based therapy with islet cells transplantation offers the possibility of physiologic glycemic control. Indeed, there are reports that islet transplantation can successfully restore long-term endogenous insulin production and glycemic stability in subjects with type I diabetes mellitus and some severe cases of pancreas fibrosis [3]. However, this method is not available for the general population of diabetes patients due to the shortage of donor cells and the requirement of long-term immunosuppression. The generation of hiPSCs [4], particularly the urine cells originated hiPSCs from the patient [5, 6], brings about an alternative choice for preparing the insulin-producing cells as seed cells for transplantation. In the last decade, several studies have reported the generation of insulin-producing cells from hiPSCs or human embryonic stem (hES) cells *in vitro* [7–12]. Kunisada (2012) reported that the differentiation efficiency of insulin-positive cells was about 10% [11], which was ranging from 10% to 15% in several experiments. Even many improvements have been made for protocols of generating insulin-producing cells from hiPSCs / hES cells, the differentiation efficiency was still very low (or far from satisfaction). Therefore, further explorations from different angles are necessary to understand the molecular basis and improve the differentiation efficiency of insulin-positive cells.

Pancreatic duodenal homeobox-1 (PDX1) is a transcription factor that is expressed in β and delta cells of the islets of Langerhans and in dispersed endocrine cells of the duodenum. It is involved in regulating the expression of a number of key β cells genes [13]. During the generation of insulin-producing cells from hiPSCs or hES cells, the process of deriving PDX1-positive pancreatic progenitors from definitive endoderm, which gives rise to the pancreas, seems to be a critical step.

Previous reports demonstrated that T has a direct impact on insulin content in the rat, and T administration can protect pancreatic β cells from chemical- induced diabetes [14]. The low serum T level was associated with insulin resistance in men and T replacement therapy reduced insulin resistance and improved glycemic control in hypogonadal men with type 2 diabetes [15, 16].

Hyperandrogenism, hyperinsulinemia and hypoglycemia are clustered symptoms present in several genetic diseases including the polycystic ovarian syndrome (PCOS), congenital adrenal hyperplasia (CAH) and Wermer's syndrome. The outcomes from clinical treatments suggest that hyperandrogenism, hyperinsulinemia and hypoglycemia are inter-linked. PCOS is a common and heterogeneous disorder occurring in women of reproductive age. It is characterized by hyperandrogenism and associated hyperinsulinemia [17]. PCOS is considered a complex multigenic disorder. However, a single-gene mutation on 11 β-hydroxysteroid dehydrogenase type 1 causes CAH which also can produce the same phenotypes of PCOS and the definitive differentiation diagnosis between PCOS and CAH is dependent on gene sequence analysis [18]. These clinical implications indicate that T may have certain relevance with the development of insulin-producing cells.

We also observed a young male case with Wermer's syndrome. This patient was initially noticed with hypoglycemia and hyperinsulinemia with pancreatic tumors [19]. Surprisingly, the laboratory tests showed that this patient also had elevated T (27.03 nM; reference range, 14~5.4 nM).

Accordingly, we hypothesize that hyperinsulinemia is secondary to the hyperandrogenism in some degree, and administration of T may improve the differentiation efficiency of insulin-producing cells from hiPSCs. To test this hypothesis, we selected three human hiPSCs lines generated from urine-derived cells (UCs), including two male samples and one female sample (UC-013, UC-015, UC-041) [20]. Ultimately, several independent trials confirmed our hypothesis. Indeed, after optimizing the conditions in our protocol, we obtained a relative higher differentiation efficiency of insulin-producing cells from hiPSCs compared with previous protocol. Furthermore, during the β cells differentiation process, we found that the expressions of key genes determining β cell and its progenitor cell lineage were increased by the formula with T administration.

## Materials and methods

### Cell culture

hiPSCs were from South China Institute for Stem Cell Biology and Regenerative Medicine Key Laboratory of Regenerative Biology, Chinese Academy of Sciences (Guangzhou, China). Cells were cultured with mTeSR1 (Invitrogen). Cells were passaged at 1:3 with accutase (Sigma) and cultured for 24h in primate mTeSR1 medium containing 5 μM Y-276342 (Sigma). Culture was carried out at 37°C under 5% CO_2_ in air. For differentiation the dissociated cells were plated into Matrigel (BD Bioscience) 1:100-coated dishes for attachment with a coverage of 70% and then incubated with RPMI1640 medium (Invitrogen) containing 100 ng/ml activin A (Peprotech), 1% B27 minus insulin (Invitrogen), 3 μM CHIR99021 (Stemgent) for 24h, and then in RPMI 1640 medium containing 100 ng/ml activin A, 1% B27 minus insulin for 48h. Subsequently, the medium was replaced with DMEM/F-12 containing 1% B27 (Invitrogen) 1 μM dorsomorphin (Calbiochem) 2 μM retinoic acid (Sigma), 10 μM SB431542 (Sigma). Cells were cultured for seven days and then these media were replaced with DMEM/F-12 medium supplemented with 1% B27. To enhance differentiation into insulin-producing cells, 10 μM forskolin (Stemgent), 10 μM dexamethasone (Enzo life sciences), 5 μM RepSox (selleckchem), 10 mM nicotinamide (STEMCELL Technologies), 2 nM T (Sigma) that enhances the efficiency of insulin content were added to the medium, DMSO (dimethylsulfoxide, 0.1% adding to the medium) as control group, and two thirds of the medium was changed with fresh one every three or four days.

### Immunocytochemistry staining

The sample cells were fixed in 1% paraformaldehyde and blocked with 3% BSA and 0.2% triton X-100 in PBS, and then incubated with primary antibody over night at 4 ^o^C and further incubated with secondary antibody (Alexa Fluor 488-or 568-conjugated donkey or goat antibodies directed against mouse, goat, rabbit or guinea pig IgG at 1:400 dilutions). The used primary antibodies induce anti-FOXA2 (rabbit IgG, 1:200, GeneTex), anti-SOX17 (mouse IgG, 1:200, R&D Systems), anti-PDX1 (goat IgG, 1:300, R&D Systems) and anti-insulin (guinea-pig IgG, 1:300, Dako). Nuclei were stained with DAPI (4,6-diamidino-2-phenylindole). Images were captured using the LEICA DMI6000B and the positive cells were counted and analyzed by Fiji imaging software [21].

### Semi-quantitive real time PCR

Total RNA was extracted using TRIzol and 1 μg RNA was used to synthesize cDNA with ReverTra Ace® (Toyobo) and oligo-dT (Takara). qPCR was performed on Bio-Rad CFX96 PCR machine with the SYBR Green Premix Ex Taq (Takara) kit. Glyceraldehyde-3-phosphate dehydrogenase (*GAPDH*) expression was used for normalization and all measurements were performed in triplicate. Related primers are listed as following: *NGN3* (137 bp), forward primer, 5’-GCGCAATCGAATGCACAACCTCAA-3’, reverse primer, 5’-TTCGAGTCAGCGCCAAGATGTAGTT-3’; *NEUROD1* (179 bp): forward primer, 5’-GGATGACGATCAAAAGCCCAA-3’, reverse primer, 5’-GCGTCTTAGAATAGCAAGGCA-3’; *INS* (67bp) forward primer, 5’-GCAGCCTTTGTGAACCAACAC-3’, reverse primer, 5’- CCCCGCACACTAGGTAGAGA-3’ and *GAPDH* (197 bp), forward primer, 5’-GGAGCGAGATCCCTCCAAAAT-3’, reverse primer, 5’-GGCTGTTGTCATACTTCTCATGG-3’. PCR was performed as denaturation for 3 minutes at 95 ^o^C, then 95 ^o^C 10 seconds, 60 ^o^C 30 seconds; for 40 cycles.

### Flow cytometry

About 10^6^ differentiated cells were dissociated into single-cell with 0.25% trypsin or accutase at 37 ^o^C. Intracellular antibody staining was performed using Becton Dickinson Cytofix/Cytoperm and Becton Dickinson Perm/Wash buffer according to manufacturer instructions. The following concentrations of primary and secondary antibodies were used: anti-FOXA2 (rabbit IgG, 1:500, GeneTex); anti-SOX17 (mouse IgG, 1:500, R&D Systems); anti-PDX1 (goat IgG, 1:500, R&D Systems); anti-insulin (guinea-pig IgG, 1:500, Dako); donkey anti-rabbit-Alexa 568,1:1000 (Invitrogen, Carlsbad, CA, USA), donkey anti-mouse-Alexa 488, 1:1000 (Invitrogen), donkey anti-goat-Alexa 488,1:1000 (Invitrogen) and goat anti-guinea pig-Alexa 488,1:1000 (Invitrogen). The cells were then washed with FACS buffer (PBS contains 2% fetal bovine serum, FBS). Control samples were stained with isotype-matched control antibodies. The cells were washed and resuspended in FACS buffer and then processed for analysis on FACS Accuri C6 (BD) or FlowJo software.

### Insulin secretion assay by ELISA

Differentiated cells were pre-incubated for 2h at 37°C in Krebs-Ringer bicarbonate HEPES buffer (KRBH:116 mM NaCl, 4.7 mM KCl, 2.5 mM CaCl_2,_ 1.2 mM KH_2_PO4, 1.2 mM MgSO_4_, 24 mM HEPES, 25 mM NaHCO_3_, and 0.1% BSA). Then, the cells were incubated for 1h at 37°C in KRBH containing the 2.5 mM D-glucose or 27.5 mM D-glucose or 30 mM potassium chloride (KCl). The human insulin levels in culture supernatants were measured with a Human Insulin ELISA kit (Millipore) according to the manufacturer's instructions. The total protein content was determined with a BCA Protein Assay Kit (Millipore) and served as internal control between groups.

### Statistical analyses

All the experiments were performed three times. Data are shown as the mean ± SD. Data was analyzed with the Student t-test. P values of less than 0.05 were considered statistically significant (*), P values of less than 0.01 were considered statistically significant (**).

## Results

### Optimized formula with T for the differentiation of insulin-producing cells from hiPSCs

**Fig 1:**
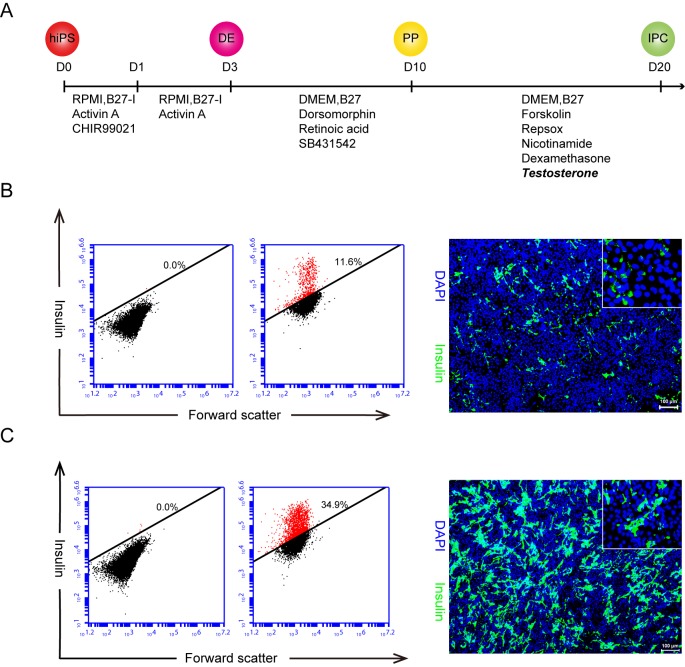
hiPSCs differentiated into insulin-producing cells by an optimized protocol. A. The schematic outlines our modified hiPSCs differentiating to insulin-producing cells protocol with key time points and components added stages, 2 nM T was added from day 10 to day 20. hiPS: human induced pluripotent stem cells; DE: definitive endoderm; PP: pancreatic progenitor; IPC: insulin-producing cells. B. The control group, none T, but same volume of DMSO was added; C. Experiment group with T administration. The flow cytometry analysis revealed at day 20 that the rate of these insulin positive cells in the control group was 11.6% (B. left panel) versus the one of 34.9% in the experiment group (C. left panel); and this data was confirmed by the insulin antibody immunostaining (showed at the right panel of B and C) and the inserted magnifying image shows the clear cytoplasmic location of insulin. Similar results were obtained in at least three independent experiments. Scale bar = 100 μm.

Three hiPSCs [UC-015 (Male), UC-013 (Male), UC-041 (Female)] generated from human urine-derived cells under feeder-free, virus-free, serum-free condition and without use of oncogene c-MYC [20] were selected in this study. We have developed a highly efficient small molecular protocol with T to direct insulin-positive cells differentiation from the hiPSCs (Fig 1A). Due to the hiPSCs having already adapted to serum-free culture, we initially chose another improvement method for generating definitive endoderm from hiPSCs so that we achieved higher efficiency of definitive endoderm with serum-free condition [22], in which activin A and GSK3β specific inhibitor CHIR99021 were utilized to induce definitive endoderm formation. At the same time, we checked the expression of FOXA2 and SOX17 to confirm the definitive endoderm by immunostaining and flow cytometry analysis, and the data showed that at day 3 of the inducing procedure, it comprised over 90% FOXA2- and SOX17-positive cells (S1A Fig). Secondly, the definitive endoderm cells were treated with retinoic acid, dorsomorphin, the BMP type I receptors (ALK2, ALK3, and ALK6) inhibitor and SB431542, the TGF-β type I receptor inhibitor to induce pancreatic progenitor and the percentage of PDX1 positive cells was more than 90% at day 10 (S1B and S1C Fig).

Several lines of evidence have shown that T administration in different animal models have improving effects of β cell surviving under toxin, insulin content and secretion [14, 16, 23]. Therefore we would like to test whether administration of T may improve the efficacy of pancreatic progenitor cells differentiating into insulin-producing cells at the differentiation stage (Day 10 to Day 20).

Thus, we focus on this stage and began to treat the cells with T at day 10 while these cells were at pancreatic progenitor stage. As control groups, we also treated the cells with T at hiPSCs stage (day 0), definitive endoderm stage (day 3), but failed to increase differentiation efficiency at day 20 (data not shown). After determined the best exposure time and period, we further tested the dose curve of T administration. We chosen the T concentration region from 1 nM to 10 nM based on two references; one is the physiological threshold of T in adult male (10.4–24.3 nM); and the other is a Wermer's syndrome case who had hypoglycemia, hyperinsulinemia and hyperandrogenemia (27 nM, Table 1). We found that 2nM T gave rise to 35% of insulin-producing-cell at day 20 (S2B Fig).

**Table 1:** A laboratory hormones test of a male Wermer's syndrome patient.

Parameter	Value	Reference
Follicle-stimulating hormone (FSH)	10.53	1.5~12.4 IU/l
Luteinizing hormone (LH)	9.06↑	1.8~8.6 IU/l
Estradiol (E2)	152.8↑	36.76~147.04 pM
Testosterone (T)	27.03↑	14~25.4 nM
Prolactin (PRL)	26.58↑	4.0–15.2 ng/ml
Parathyroid hormone (PTH)	219.3↑	15–65 pg/ml

↑: The arrow indicates higher value than the reference.

Finally, we finalized our protocol that adding 2nM T from day 10 to day 20 which is the stage of the pancreatic progenitor differentiating to insulin-producing cells (Fig 1A), and other components are similar as described in the originated report [11] Compared with the previously reported efficiency of 10% for insulin-positive cells (Fig 1B) as assayed by flow cytometry analysis [11], the new induction formula with T improved the differentiation efficiency of insulin-producing cells more than 3 times (Fig 1C). The average of the differentiation efficiency of these three tested hiPSCs was 34.6% (S2A Fig). Our optimized approach resulted in much higher efficiency.

### Analysis of gene expressions related to pancreatic β cells fate and the insulin secretion response test

**Fig 2:**
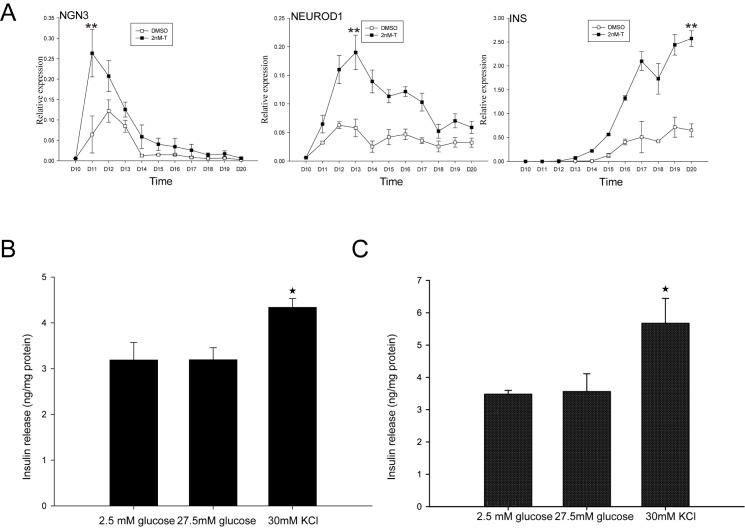
T administration upregulates the expression of pancreatic β cells fate-associated genes and insulin secretion assay. A. Transcript levels for different pancreatic genes during final differentiation stage. All results shown are from three or more independent experiments. Error bars indicate s.d. **P<0.01 by Student‘s t-test. B. Insulin secretion of the T administrating group; C. Insulin secretion of control group; day 20 insulin-producing cells in response to D-glucose and potassium chloride (30 mM). Error bars indicate s.d. *P<0.05 by Student‘s t-test.

On the day 10 of this protocol, the growth of PDX1-positive cells was robust (S1B Fig). At this time point, the T was added to the culture medium in different concentrations till the end of the insulin-producing cells differentiation and the maturation, the same amount of DMSO was added in both experiments group and control group. To search for the clue of T administration promoting the change of the β cell fate, we examined mRNA expression of three land marker genes during the process. The semi-quantitive real time PCR was performed on pancreatic β cells-specific transcription factors and marker genes (*NGN3*, *NEUROD1*, *INS*) (Fig 2A). Starting from Day 10, these PDX1 positive cells were exposed to the medium with/without T, the *NGN3* expression quickly peaks at Day 11 and extended into Day 14. *NGN3* expression were present in both groups and the pattern of the time curves was similar, but the *NGN3* expression level of T groups nearly doubled to the control groups (Fig 2A); sequentially, the *NEUROD1* expression peaked at Day 14 and extended into Day 18 (Fig 2A); The expression of insulin mRNA was analyzed from day10 to day20; the expression were starting from day 13 and gradually reached the peak at Day 20 (Fig 2A). However, the related genes expressions with T groups were increased 4 fold (*NEUROD1*) to 5 fold (*INS*). These data indicate that androgen plays a certain role in promoting the PDX1 positive cells toward the fate of insulin-producing cells. To further assess whether the insulin secretion of these T administration induced insulin-positive cells have physiological response to stimulations, we measured the insulin level in the supernatant after incubation with glucose and potassium chloride (KCl). Glucose stimulated insulin secretion is one of the most important physiological function indicator of pancreatic β cells. Although the standard high glucose stimulation did not increase insulin secretion, the KCl stimulation lead to significantly increased insulin secretion, as expected (Fig 2B). The partial responses for those physiological stimulations suggest that the insulin-producing cells are not fully matured β cells. In terms of the physiological response of the insulin secretion, it is still a field to be further studied. The control group has the similar secretion responses results (Fig 2C).

## Discussion

Our experiments demonstrate that T enhances the differentiation efficiency of hiPSCs to insulin-producing cells and it is not gender dependent. The original idea came from the observation of a clinical case with multiple endocrine neoplasia type1 (MEN1) or Wermer's syndrome. A young male inherited a single nucleic acid mutation on the exon 9 of the *MEN1* gene from his mother, and his symptoms initially noticed were dizziness and difficult to rouse in the morning. Moreover, the patient found that these symptoms can be improved by consuming a large amount of sugar. Laboratory results also indicated that there are co-existed hyperinsulinemia and hyperandrogenism along with the hypoglycemia. During the process of establishing the MEN1 model from urine cells originated hiPSCs and molecular mechanism study, a speculation came up that there may be a relationship between T and insulin.

Encouraging by the clues from two genetic diseases, PCOS and CAH, in which hyperandrogenism and hyperinsulinemia are simultaneously co-existed, therefore we attempted to test whether the T play a role for the pancreatic progenitor cell differentiating to insulin-producing cells. One outcome from these experiments established a new formula for pancreatic β cells differentiation that dramatically increased the insulin-producing cells fate comparing to these routine protocols. One potential mechanism is that T enhances PDX1 positive progenitor cells sequentially expressing genes associated with pancreatic β-cell fate decisions.

Previous research showed that the progenitors of pancreatic endocrine cells transiently express the transcription factor Neurogenin3 (NGN3) which belongs to basic helix-loop-helix (bHLH) family. Lineage tracing demonstrated that all islet endocrine cell types are derived from NGN3-positive cells. Therefore, in vitro induction of *NGN3* expression in hiPSCs-derived pancreatic PDX1 positive progenitors is essential to generate β cells [24, 25]. During the development of pancreatic endocrine, NGN3 protein directly binds to the promoters of the pancreatic β cells specific transcription factors β2/NEUROD1 and Pax4 to promote the differentiation of PDX1-progenitor cells into the pancreatic β cells lineage [26]. Our data also revealed that administration of T sequentially up-regulated the expressions of *NGN3* and *NEUROD1* that gave raise the rate of insulin-producing cells fate. Therefore, during embryogenesis, in particular of the pancreatic organogenesis, when and what kind of cell type(s) express androgen receptor(s) are worth to be further dissected out. The detailed mechanistic linkage between the pancreatic β cells lineage and androgen signaling will help further optimize protocols for *in vitro* guiding pancreatic β cells fate from multipotent stem cells; furthermore, seed cells generated from an optimized fully defined small molecule formula will benefit the future applications in regenerative medicine and pharmaceutical production of insulin based on human insulin producing cells.

The role of androgen for pancreas development is still an open question; however, certain roles of androgen in pancreatic physiology and metabolism of the body are clearly demonstrated. Several studies have already demonstrated that T can significantly decrease early apoptotic damage induced by streptozotocin in rat pancreas [27, 28]. Inadequate levels of androgens are associated with an increased risk of obesity and diabetes. Navarro et al. identified that androgen receptors were located in pancreatic β cells, and mediated the enhancement of glucose-stimulated insulin secretion in the male by increasing islet cAMP and activating the PKA, in particular improving the insulin resistance of type 2 Diabetes with hypo-T [29]. More than 43 clinical trials were/are ongoing to test T therapy of men with type 2 Diabetes [30]. It already has positive data that shows T treatment increase lean body mass and lipid oxidation as well as insulin sensitivity in hypogonadal men [31].

The physiological threshold of plasma T (free or bound) is ranged from 10.4–24.3 nM in adult men, and 0.35–2.6 nM in women [32]. The highest concentration of T used in our experiments is 10nM that is in the normal range of adult men; moreover, the optimal concentration of T is 2 nM which give rise to the highest rate of insulin-producing cells fate (S2B Fig).

At this stage, even though higher differentiation efficiency was achieved, in term of the insulin secretion, our insulin-producing cells just had partially response to physiological stimulations (Glucose and potassium). Indeed, the expression of a key glucose transporter (GLUT2) had no significant difference between the T administration groups and the controls (S2C Fig). The shortage of GLUT2 might be one reason for the lack of fully responsive of insulin secretion stimulations. Further study optimizing the induction condition is needed.

Our hypothesis and investigation was started and encouraged by our clinic observation that hypoglycemia, hyperinsulinemia and hyperandrogenemia were co-existed in a young male case with Wermer's syndrome. In summary, this report brings three conceptual advances of related biomedical topics. First, we demonstrate that T promotes the differentiation efficiency of pancreatic β derived from hiPSCs. Second, T sequentially drives the expression of key genes associated with β cells differentiation that are the pancreatic β cell progenitor master genes: *NGN3*, *NEUROD1* and *INS* (the key genes of β cell). To our knowledge, this is the first time that this mechanism is reported. Third, we obtained a higher efficient method to induce insulin-producing cells from hiPSCs by a fully defined small molecular formula, and the efficacy is 3 fold higher comparing to these routine protocols. The detailed underlying mechanisms, such as the pathways and genes play pivotal roles in this story are ongoing investigation to dissect out though multi-omics analyses and lost/gain function manipulations.

## Supporting information

S1 Fig Detection of specific protein expression. A. Cells were immunostained with SOX17, FOXA2 antibodies on day 3. Flow cytometry analysis of differentiated hiPSCs revealed that 95.4% of cells were SOX17- and FOXA2-double-positive cells. Scale bar = 100 μm. B. Cells were immunostained with PDX1 antibodies on day 10. Scale bar = 50 μm. C. Flow cytometry analysis revealed that the differentiated hiPSCs comprised 92.3% PDX1-positive cells at the day 10.
(EPS)

S2 Fig Analyses of insulin-producing cells treated with T. A. The percentage of insulin-positive cell derived from three kinds of hiPSCs by flow cytometry; CTR: the control samples were stained with isotype-matched control antibodies, as a negative group in flow cytometry experiment. DMSO: administration of the DMSO control group. 2 nM T: administration concentration of T is 2 nM as the experiment group. B. Percentage of insulin-producing cells relationship with different concentration of T. The results are representative of three independent experiments Error bars indicate s.d. **P<0.01 by Student‘s t-test. C. GLUT2 expression level for different pancreatic genes during final differentiation stage.
(EPS)
